# A spatio-temporal model of multi-marker antimalarial resistance

**DOI:** 10.1098/rsif.2023.0570

**Published:** 2024-01-17

**Authors:** Yong See Foo, Jennifer A. Flegg

**Affiliations:** School of Mathematics and Statistics, The University of Melbourne, Parkville, Australia

**Keywords:** antimalarial drug resistance, spatio-termporal mapping, sulfadoxine-pyrimethamine, haplotype frequency estimation, latent multinomial

## Abstract

The emergence and spread of drug-resistant *Plasmodium falciparum* parasites have hindered efforts to eliminate malaria. Monitoring the spread of drug resistance is vital, as drug resistance can lead to widespread treatment failure. We develop a Bayesian model to produce spatio-temporal maps that depict the spread of drug resistance, and apply our methods for the antimalarial sulfadoxine-pyrimethamine. We infer from genetic count data the prevalences over space and time of various malaria parasite haplotypes associated with drug resistance. Previous work has focused on inferring the prevalence of individual molecular markers. In reality, combinations of mutations at multiple markers confer varying degrees of drug resistance to the parasite, indicating that multiple markers should be modelled together. However, the reporting of genetic count data is often inconsistent as some studies report haplotype counts, whereas some studies report mutation counts of individual markers separately. In response, we introduce a latent multinomial Gaussian process model to handle partially reported spatio-temporal count data. As drug-resistant mutations are often used as a proxy for treatment efficacy, point estimates from our spatio-temporal maps can help inform antimalarial drug policies, whereas the uncertainties from our maps can help with optimizing sampling strategies for future monitoring of drug resistance.

## 1. Introduction

Malaria is a deadly disease caused by parasites that are transmitted by mosquitoes. During the treatment of a malaria infection, the parasites undergo selective pressure, favouring the survival of parasites that have genetic mutations which confer on them resistance against the drug treatment. For *Plasmodium falciparum*, the most common species of malaria parasites, drug resistance is a major threat to the control of the disease. It is therefore important to be able to quantify changes in antimalarial drug resistance, including for sulfadoxine-pyrimethamine (SP) that is used for intermittent preventive treatment for high-risk groups, namely pregnant people and young children.

Spatio-temporal trends in antimalarial drug resistance can be monitored using molecular markers known to be associated with drug resistance as a proxy of clinical efficacy. *Plasmodium falciparum* resistance against SP is characterized by mutations on the *dhps* and *dhfr* genes [1]. In fact, SP-resistant parasites often carry multiple SP-resistant mutations (a set of molecular markers or a haplotype). Genetic studies are easier to conduct and are a fraction of the cost of a clinical study—allowing for larger numbers of samples to be collected across more spatio-temporal locations [2]. This makes data from genetic studies readily amenable to model-based geostatistics.

To our knowledge, all works to date that have statistically mapped the geospatial distribution of the *dhps* and *dhfr* markers have modelled each marker separately [3–6]. Most relevant to the work presented in our paper, Flegg *et al.* [3] developed a predictive model for the geographical and temporal trends across Africa of the prevalence of mutations on the *dhps* gene of the parasite that are associated with SP resistance. A separate model was used for each marker (*dhps* A437G, *dhps* K540E and *dhps* A581G), which models the count data with binomial distributions. Correlation between binomial probabilities are specified according to the spatio-temporal distance between their corresponding sites. Specifically, the logit transformation of the binomial probabilities are set to follow a Gaussian process (GP) distribution.

Although existing spatio-temporal mapping work has focused on individual marker mutations, molecular studies have shown that it is the presence of the double mutant haplotype *dhps* A437G/K540E and triple mutant haplotype *dhps* A437G/K540E/A581G in the parasite that are most strongly associated with an increased risk of SP treatment failure [7,8], and thus the most clinically relevant. Therefore, new modelling approaches are needed to obtain spatially continuous maps of haplotype prevalences, not just of individual marker mutation prevalences. However, not all studies report the presence or absence of each mutation simultaneously, i.e. the counts of full haplotypes are not reported. Instead, studies may only report the number of samples that carry each individual marker mutation. Since the samples corresponding to each reported count may overlap, we cannot use a multinomial distribution directly. Moreover, some studies only report on a smaller subset of all mutations of interest. We handle these discrepancies caused by partially reported data under a *latent multinomial model* [9,10], where the observed counts are treated as binary combinations of unobserved multinomial counts. In this paper, we extend the spatio-temporal GP models of individual *dhps* markers [3,6] to model the prevalences of multiple haplotypes by using a latent multinomial distribution with GPs. Handling all haplotypes within one model allows us to leverage all available data to greater utility.

This paper is structured as follows. In §2, we present the latent multinomial GP model for mapping SP drug resistance based on mutations on the *dhps* gene. We do not account for mutations on the *dhfr* gene as mutations on the *dhps* gene are more closely related to clinical SP failure and there is a triple *dhfr* mutation widely spread across Africa already [11]. This is followed by the outputs of the model in §3, showing the prevalence of each haplotype of interest over space and time. Finally, in §4, we discuss the implications of our findings.

## 2. Methods

In this paper, we construct a Bayesian hierarchical model that is capable of modelling partially reported multinomial count data for spatio-temporal changes in drug resistance. This modelling framework is needed to handle reporting inconsistencies found across studies on the prevalences of drug-resistant haplotypes, where different studies may report on different combinations of mutations. For example, the list of *dhps* mutations compiled by the Worldwide Antimalarial Resistance Network [12] includes: *dhps* 437G, *dhps* 540E, *dhps* 581G, *dhps* 437G-540E, *dhps* 437G-540E-581G and *dhps* 437G-540E-A581. These inconsistencies in data collection and study design significantly complicate model construction and parameter inference. We develop a latent multinomial model (§2.1) with a GP prior specification (§2.2). We describe how spatio-temporal maps of haplotype prevalences are produced under a Bayesian framework in §2.3.

### 2.1. Latent multinomial model formulation

Suppose that we have *G* molecular markers of interest for monitoring drug resistance (table 1). For our application, we have *G* = 3 molecular markers, namely *dhps* 437, *dhps* 540 and *dhps* 581. Define a *full haplotype* to be a binary string of length *G* recording whether each of these mutations are present (1) or absent (0). This results in *H* = 2^*G*^ possible full haplotypes.

**Table 1.** Summary of notation used in §2.1.

notation	description
*G*	no. molecular markers under consideration
*H*	no. full haplotypes under consideration
*N*	no. data points
*R*_*i*_	no. realized haplotypes that data point *i* reports
**p**_*i*_	vector (length *H*) of full haplotype probabilities at data point *i*
**z**_*i*_	vector (length *H*) of full haplotype counts at data point *i*
**y**_*i*_	vector (length *R*_*i*_) of realized haplotype counts at data point *i*
**A**_*i*_	binary matrix (size *R*_*i*_ × *H*) linking realized haplotypes to full haplotypes

**Figure 1.**
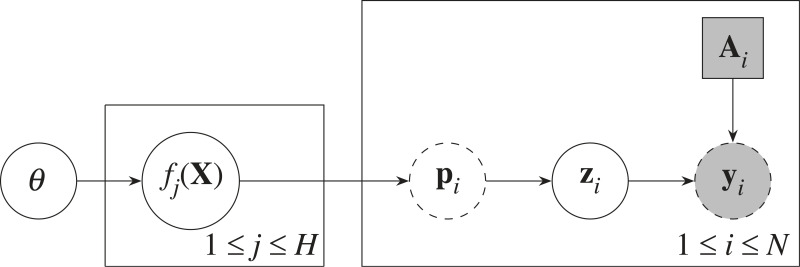
Graphical model for latent multinomial data with multiple populations whose haplotype prevalences ${\left\{p_{i}\right\}}_{i=1}^{N}$ are correlated through Gaussian processes. *f*_*j*_(**X**) denotes the vector (*f*_*j*_(**x**_1_), …, *f*_*j*_(**x**_*N*_)). Circles correspond to random variables while squares correspond to constant values. A shaded node indicates that the variable is observed. A dotted outline indicates that the variable is deterministically calculated from its parent variables. Variables contained within a plate (box) are replicated according to the index at the bottom right.

Over space and time we have *N* studies (figure 1); study *i* (*i* = 1, …, *N*) is conducted to estimate the prevalence **p**_*i*_ at this location of the *H* haplotypes associated with drug resistance. We define **z**_*i*_ to be a vector of length *H* that stores the number of each of the *H* full haplotypes for the *i*th study, and *n*_*i*_ = *z*_*i*,1_ + *z*_*i*,2_ + · · · + *z*_*i*,*H*_ to be the known sample size of the study. The counts ${\left\{z_{i}\right\}}_{i=1}^{N}$ are considered unobserved (latent), each following a multinomial distribution:

2.1 $$z_{i}|p_{i}∼\mathrm{Mult}(n_{i},p_{i})\;\text{for }i=1,\ldots ,N.$$

For each *i* = 1, …, *N*, we observe binary combinations of the latent counts, depending on the way counts are reported in the *i*th study. We refer to the set of full haplotypes that are included by a single observed count as a *realized haplotype*. For example, the realized haplotype 437G-540E includes the full haplotypes 110 (437G-540E-A581) and 111 (437G-540E-581G). The collection of realized haplotypes reported may vary across studies, which we encode within a *configuration matrix*. As an example, for a study which reports on the realized haplotypes *dhps* 437G, *dhps* 540E, *dhps* 581G, *dhps* 437G-540E, *dhps* 437G-540E-A581 and *dhps* 437G-540E-581G, we have the following configuration matrix:

2.2 $$\begin{array}{rl} \begin{array}{c} \;\;000\;001\;010\;011\;100\;101\;110\;111 \end{array} & \\ \begin{array}{c} \;dhps\;437G\\ \;dhps\;540E\\ \;dhps\;581G\\ \;dhps\;437G-540E\\ \;dhps\;437G-540E-581G\\ \;dhps\;437G-540E-A581 \end{array}\left[\begin{array}{cccccccc} 0\; & 0\; & 0\; & 0\; & 1\; & 1\; & 1\; & 1\\ 0\; & 0\; & 1\; & 1\; & 0\; & 0\; & 1\; & 1\;\\ 0\; & 1\; & 0\; & 1\; & 0\; & 1\; & 0\; & 1\;\\ 0\; & 0\; & 0\; & 0\; & 0\; & 0\; & 1\; & 1\;\\ 0\; & 0\; & 0\; & 0\; & 0\; & 0\; & 0\; & 1\;\\ 0\; & 0\; & 0\; & 0\; & 0\; & 0\; & 1\; & 0\; \end{array}\right] \end{array}.$$

In this way, for an observed count of the samples with the *dhps* 437G mutation, we need to sum the number of the four haplotypes {100, 101, 110, 111}, while for an observed count of the samples with dhps 437G-540E, we only need to sum the haplotypes {110, 111}. The configuration matrix is constructed based on which haplotypes are included in each count reported by a study.

We denote the number of realized haplotypes reported in study *i* by *R*_*i*_ and the reported counts of the realized haplotypes by vector **y**_*i*_ of length *R*_*i*_, for *i* = 1, …, *N*. Let **A**_*i*_ be the constructed *R*_*i*_ × *H* configuration matrix of 0’s and 1’s, determined from the realized haplotypes reported in study *i*. For each *i* = 1, …, *N* (figure 1), the latent counts **z**_*i*_ must be a non-negative integer solution to the system

2.3 $$y_{i}=A_{i}z_{i}$$

and the sample size constraint *n*_*i*_ = *z*_*i*,1_ + *z*_*i*,2_ + · · · + *z*_*i*,*H*_. Since the sample size, *n*_*i*_, is known, we let the first row of **A**_*i*_ always be a vector of ones and the first observed count be the sample size, i.e. *y*_*i*,1_ = *n*_*i*_ for all *i*.

To calculate the likelihood, we marginalize out the latent counts ${\left\{z_{i}\right\}}_{i=1}^{N}$ exactly by enumerating all possible latent counts **z**_*i*_ that satisfy (2.3). The full likelihood is

2.4 $$p(y_{1},\ldots ,y_{N}|p_{1},\ldots ,p_{N})=\prod_{i=1}^{N}p(y_{i}|p_{i}),$$

where

2.5 $$\begin{array}{rl} \;p(y_{i}|p_{i}) & =\sum_{z_{i}\;:\;A_{i}z_{i}=y_{i}}p(y_{i},z_{i}|p_{i})\\ & =\sum_{z_{i}\;:\;A_{i}z_{i}=y_{i}}\underset{p(z_{i}|p_{i})}{\underbrace{\begin{array}{c} n_{i}\\ z_{i1},\ldots ,z_{iH} \end{array}p_{i1}^{z_{i1}}\cdots p_{iH}^{z_{iH}}}}\underset{p(y_{i}|z_{i})}{\underbrace{1(y_{i}=A_{i}z_{i})}}. \end{array}$$

The summation in (2.5) requires us to enumerate, for each *i* = 1, …, *N*, the *feasible set* of solutions

2.6 $$F(A_{i},y_{i})\;:=\{z\in Z_{\geq 0}^{H}\;:\;A_{i}z=y_{i}\},$$

where $Z_{\geq 0}^{H}$ is the space of *H*-dimensional non-negative integer vectors. We find all elements of the feasible set with a branch-and-bound algorithm (electronic supplementary material, S1). This algorithm is run once prior to parameter inference.

### 2.2. Gaussian process prior specification

To account for the correlation between haplotype prevalences for different studies, we model the haplotype prevalences as a softmax transformation of *H* independent GPs:

2.7 $$\begin{array}{rl} & p_{ij}=\frac{\exp(f_{j}(x_{i}))}{\exp(f_{1}(x_{i}))+\cdots +\exp(f_{H}(x_{i}))}\\ & \;\text{for }i=1,\ldots ,N,\;j=1,\ldots ,H \end{array}$$

and

2.8 $$\begin{array}{rl} & f_{j}(x_{1}),\ldots ,f_{j}(x_{N})∼N(m_{j}(X),C_{j}(X,X))\;\text{for }j=1,\;\ldots ,H, \end{array}$$

where **p**_*i*_ are the haplotype prevalences of population *i*, $X={\left\{x_{i}\right\}}_{i=1}^{N}$ are the spatio-temporal coordinates and covariates for each study, and *f*_*j*_ is the *j*th GP whose mean function and covariance function are *m*_*j*_ and *C*_*j*_ respectively. The vector *m*_*j*_(**X**) denotes the concatenation of (*m*_*j*_(**x**_1_), …, *m*_*j*_(**x**_*N*_)), and *C*_*j*_(**X**, **X**) is a matrix whose (*i*, *i*′)th entry is *C*_*j*_(**x**_*i*_, **x**_*i*′_). The mean and covariance functions are further parametrized by GP hyperparameters ***θ*** (figure 1).

For each data point *i* = 1, …, *N*, our covariates **x**_*i*_ = (*λ*_*i*_, *ϕ*_*i*_, *t*_*i*_, *r*_*i*_) consist of the longitude *λ*_*i*_, latitude *ϕ*_*i*_, median year of study *t*_*i*_, and *P. falciparum* parasite rate *r*_*i*_ (as estimated from the Malaria Atlas Project [13]). We assume that the mean function varies linearly with the parasite rate:

2.9 $$m_{j}(x_{i})=\mu_{j}+\beta_{j}r_{i},$$

where *μ*_*j*_ is a baseline mean value and *β*_*j*_ quantifies the effect of parasite rate on the prevalence of haplotype *j*. We choose our covariance function from the Gneiting class of covariance functions on a sphere [14], along with a white noise term:

2.10 $$\begin{array}{r} C_{j}(x_{i},x_{i^{\prime }})=s_{j}^{2}{\left(1+\frac{{\left(t_{i}-t_{\;i^{\prime }}\right)}^{2}}{\tau_{j}^{2}}+\frac{d_{\mathrm{GC}}(x_{i},x_{i^{\prime }})}{\delta_{j}}\right)}^{-1}+\;\sigma^{2}1(i=i^{\prime }), \end{array}$$

where $s_{j}^{2}$ is the spatio-temporal variance, *τ*_*j*_ is the timescale parameter, *δ*_*j*_ is the lengthscale parameter, *σ*^2^ is the noise variance, *d*_GC_(**x**_*i*_, **x**_*i*′_) is the great circle distance (in degrees) between data points *i* and *i*′, and 1(⋅) is the indicator function. Other Gneiting covariance functions are possible, but we choose a simple one that resembles the rational quadratic covariance function. Note that all hyperparameters except for *σ*^2^ are haplotype-specific. We place weakly informative priors on the GP hyperparameters $\theta ={\left\{\mu_{j},\beta_{j},s_{j},\tau_{j},\delta_{j}\right\}}_{\;j=1}^{H}\cup \{\sigma \}$; see electronic supplementary material, S2, for details.

### 2.3. Bayesian inference

We perform Bayesian inference using Markov chain Monte Carlo (MCMC) to obtain the posterior distribution

2.11 $$p(p_{1},\ldots ,p_{N},\theta |Y)\propto p(Y|p_{1},\ldots ,p_{N})p(p_{1},\ldots ,p_{N}|\theta )p(\theta ),$$

where **Y** denotes the collection of all observed data (**y**_1_, …, **y**_*N*_), the likelihood *p*(**Y**|**p**_1_, …, **p**_*N*_) is defined in (2.4) and (2.5), the prior *p*(**p**_1_, …, **p**_*N*_|***θ***) in §2.2 and the hyperprior *p*(***θ***) in electronic supplementary material, S2. We use the No-U-Turn sampler (NUTS) [15] to perform MCMC sampling, which uses gradient information of the logarithm of (2.11) to produce posterior chains of low autocorrelation. We run 5 MCMC chains each with 1000 iterations, discarding the first half as burn-in iterations. After MCMC sampling, we produce predictive maps of haplotype prevalences on grid cells of size $0.2^{\circ }\times 0.2^{\circ }$ across sub-Saharan Africa for each year between 2000 and 2020. Let p* denote the vector of haplotype prevalences for an arbitrary grid cell with covariates x*. To obtain samples from the posterior *p*(p*|**Y**), we draw samples from the distribution *p*(p*|**p**_1_, …, **p**_*N*_, ***θ***), where the values of **p**_1_, …, **p**_*N*_, ***θ*** are taken from the posterior samples output by NUTS. This is justified by the fact that

2.12 $$p(p^{*}|Y)=\int p(p^{*}|p_{1},\ldots ,p_{N},\theta )p(p_{1},\ldots ,p_{N},\theta |Y)\;dp_{1},\;\cdots ,\;dp_{N}\;d\theta .$$

Based on GP theory [16], the distribution *p*(p*|**p**_1_, …, **p**_*N*_, ***θ***) follows a normal distribution with the softmax transformation (2.7) applied, allowing the posterior predictive distribution *p*(p*|**Y**) to be exactly sampled given samples of the posterior distribution *p*(**p**_1_, …, **p**_*N*_, ***θ***|**Y**).

## 3. Results

We fit our latent multinomial GP model to SP resistance data in Africa, where it is common for parasites to have multiple SP-resistant mutations on the *dhps* gene and studies may report the number of samples with mutations differently. Here, we consider *G* = 3 mutations on the *dhps* gene, namely A437G, K540E and A581G, leading to *H* = 8 distinct full haplotypes. We use a dataset collated by the Worldwide Antimalarial Resistance Network [12], as detailed in electronic supplementary material, S3. This dataset reports a total of six realized haplotypes, listed in (2.2), although each data point typically does not report all realized haplotypes. Our primary goal is to obtain Bayesian estimates of the prevalences (i.e. multinomial probabilities) of the 8 full haplotypes across sub-Saharan Africa over the duration of interest 2000–2020.

The total computational time for preprocessing and MCMC was 7.9 h. We achieve a potential scale reduction factor [17] of $\hat{R}<1.02$ for all parameters, and each haplotype prevalence had an effective sample size greater than 500. The MCMC chains exhibit good mixing, as indicated by the representative trace plots shown in electronic supplementary material, figure S1.

**Figure 2.**
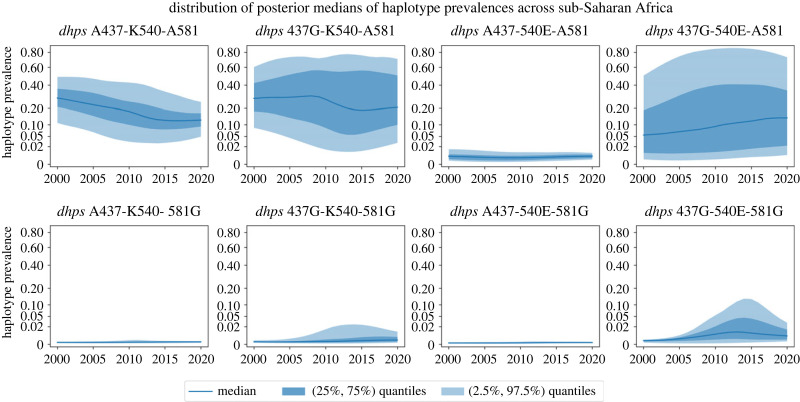
Distribution (over grid cells of study area) of the posterior medians of *H* = 8 prevalences of *dhps* haplotypes from year 2000 to year 2020. The dark blue line represents the median over the grid cells of our study area, the dark blue band represents the 50% interval and the light blue band the 95% interval. Posterior medians in all panels are presented on a square root scale.

We divide the region of interest into a $0.2^{\circ }\times 0.2^{\circ }$ grid, where we define our area of interest to be the region where the Malaria Atlas Project maps malaria transmission [13,18]. For each spatio-temporal coordinate x* of a grid cell and year, we find the posterior predictive distribution of the full haplotype prevalences at x*. Figure 2 shows the distribution of posterior median prevalences over the region of interest for all eight haplotypes during 2000–2020. Over this region, of the *H* = 8 haplotypes, three have very low prevalence over the entire duration of interest: A437-540E-A581, A437-K540-581G and A437-540E-581G.

**Figure 3.**
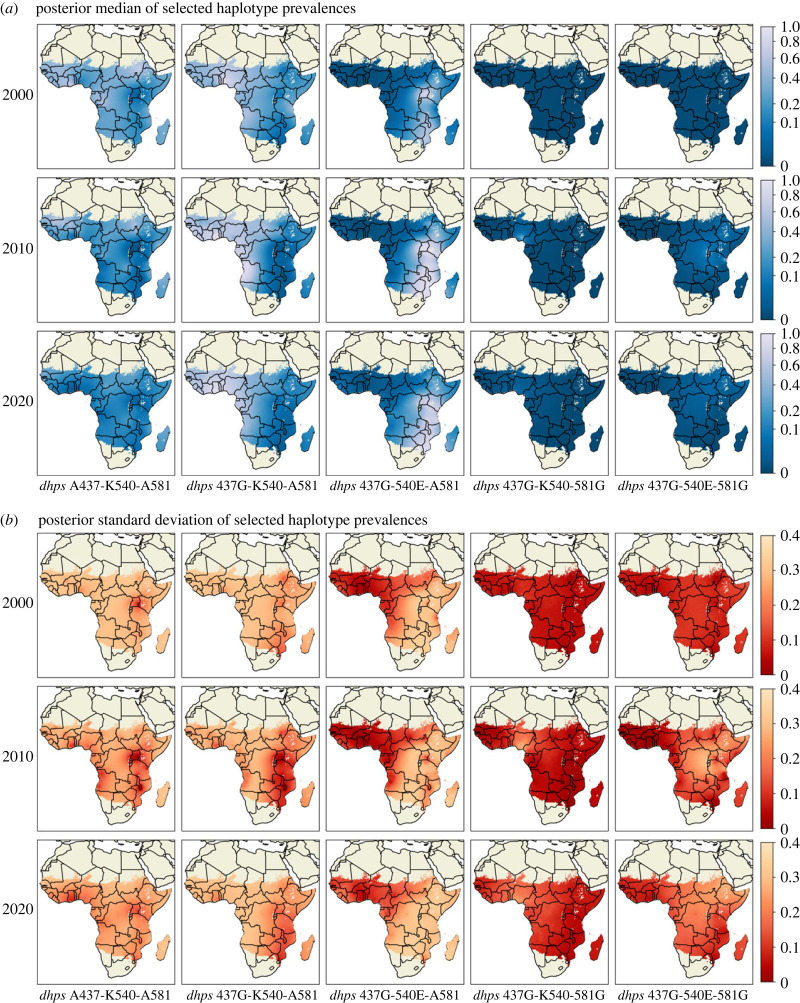
(*a*) Posterior median and (*b*) posterior standard deviation for prevalences of selected *dhps* haplotypes in years 2000 (top row), 2010 (middle row) and 2020 (bottom row).

We also provide visual summaries of the spatial distribution of prevalences of 5 selected haplotypes (the three low prevalence haplotypes from figure 2 are excluded) and their change over time. Figure 3 shows the median and standard deviation summaries of the posterior predictive distributions over the region of interest in the years 2000, 2010 and 2020. The results presented in this figure are broadly consistent with literature in that the vast majority of mutant *dhps* haplotypes have the A437G mutation [19]. The spatial patterns shown by our heatmaps agree with the results of Naidoo & Roper [20], who report the double mutation A437G-K540E as the most prevalent mutant haplotype in East Africa (third column), and also the single mutation A437G as the most prevalent mutant haplotype in West and Central Africa (second column).

**Figure 4.**
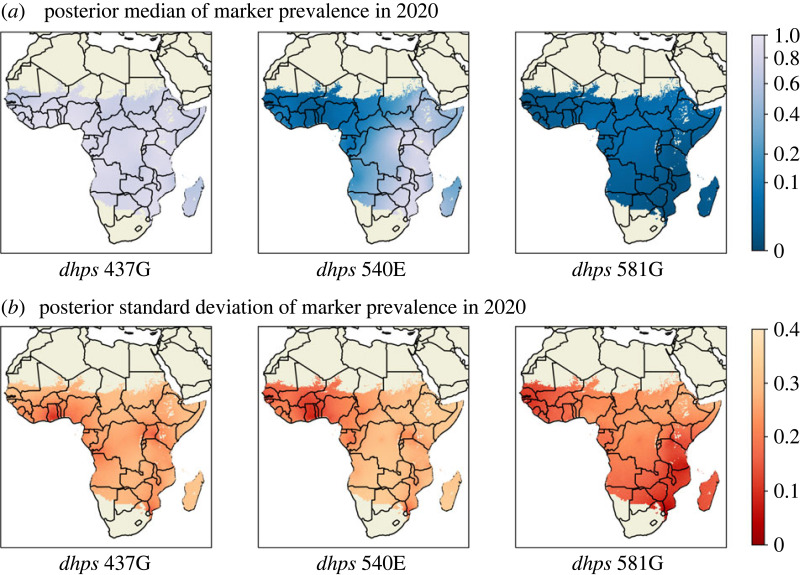
Posterior summary for prevalences of selected *dhps* individual markers in year 2020. The top row shows the posterior median for *dhps* 437, 540, 581; the bottom row shows the posterior standard deviation for *dhps* 437, 540, 581.

In the case where the prevalence of an individual mutation (A437G, K540E or A581G) is of interest, these can still be captured as outputs of our model by simply summing over the halplotypes that include the mutation. In figure 4, the spatial distributions of A437G, K540E and A581G are summarized with posterior median (top row) and standard deviation (bottom row) in 2020. These results are broadly consistent with results shown in Flegg *et al.* [6] for the same three markers in 2020.

To assess the predictive utility of our model, we rerun the inference 10 times, each time with a different 10% of the dataset withheld from inference. For each data point of the full dataset, the haplotype prevalences are predicted by the posterior median obtained from the inference instance that did not include the data point. Details of this model validation procedure are given in electronic supplementary material, S4. We report in table 2 the mean error (measure of bias) and mean absolute error (measure of average discrepancy) between predictive median and observed prevalences for each realized haplotype. Means for each realized haplotype are taken over the data points that report the count of that realized haplotype. For the realized haplotypes that involve one mutation only, we compare our errors to those obtained by Flegg *et al.* [6], who performed spatio-temporal mapping in the same study area for individual *dhps* mutations separately. Flegg *et al.* used the same 10-fold cross-validation approach as us; see table 3 in [6] for their results. Our mean absolute errors are comparable to those of Flegg *et al*. and the direction of bias (i.e. sign of mean error) concurs for all three mutations. However, the mean errors we report have larger magnitudes, possibly due to our dataset being smaller than the dataset used in [6]. Nevertheless, there is good agreement between the predictive median and observed prevalences; see electronic supplementary material, figure S2, for scatterplots comparing the predictive medians to the corresponding observed prevalences.

**Table 2.** Number of data points that report the counts for each realized haplotype, and the corresponding mean error and mean absolute error between predictive median and observed prevalences.

realized haplotype	no. data points	mean error	mean absolute error
*dhps*437G	208	0.0389	0.1158
*dhps*540E	214	0.0096	0.0731
*dhps*581G	154	−0.0302	0.0553
*dhps*437G-540E	65	−0.0060	0.0451
*dhps*437G-540E-581G	35	−0.0518	0.0778
*dhps*437G-540E-A581	33	−0.0150	0.0758

## 4. Discussion

In this paper, we develop a spatio-temporal geostatistical model to infer for the first time the prevalence of multi-marker drug-resistant malaria. We illustrate the utility of this new model for SP, which is a commonly used drug for intermittent preventive treatment of malaria in pregnancy, children and infants. Since drug-resistant haplotypes and markers are often used as a proxy for treatment efficacy, these maps can help inform antimalarial drug policies. Our methods take on a Bayesian approach, which are able to quantify uncertainties about the prevalence of the drug resistance haplotypes. A benefit of quantifying uncertainty is its use in optimizing sampling strategies for future monitoring of drug resistance [21].

Existing geostatistical methods in spatio-temporal modelling of antimalarial drug resistance use a binomial likelihood [3,6,21,22], which can only infer the prevalence of a single category, e.g. prevalence of one mutation, or prevalence of the wild-type haplotype. Our models are capable of handling multiple haplotypes simultaneously by using a multinomial likelihood, leading to more refined inference about drug resistance. This has not been done in previous work, as not all studies provide data on full haplotypes; studies may only report on a subset of mutations, or group haplotypes by the number of mutations present, or provide counts for each mutation separately. We were able to handle these types of partially reported data by using a latent multinomial model that treats each reported category as a subset of all full haplotypes. Although the counts of each full haplotype are not all experimentally determined, our approach of enumerating all possible latent counts allows us to leverage the partially reported data for inferring the prevalences of the full haplotypes.

One limitation of our work presented here is that sampling bias may be present due to population heterogeneity. For example, since SP is commonly used for intermittent preventive treatment of malaria, many studies of SP resistance take blood samples from pregnant people. Bias may also arise from the choice of mutations reported. If the prevalence of a mutation is very low, it is less likely to be reported by a study. This may lead to an overestimation in the prevalence of mutations that are not often reported. Another limitation is a lack of model checking to verify whether our model fits the data adequately. Since the haplotype categories reported are inconsistent across studies, it is not straightforward as to what model checking procedures should be applied.

A possible extension is to include more covariates for the GP model. Of biological interest are covariates related to drug pressure, such as treatment-seeking rates [23]. However, using more covariates implies that more model parameters need to be inferred. Our current MCMC approach is already computationally expensive, an issue that may be exacerbated by the inclusion of more covariates. For a dataset with more markers and/or larger pools, our enumeration approach may become infeasible, as there are too many possible latent count solutions to enumerate. In this case, we can instead treat the latent counts as model parameters to be sampled during MCMC using a custom MCMC sampler [10] based on Markov bases [24]. MCMC sampling of the latent counts cannot be performed by gradient-based samplers such as NUTS, as they cannot handle discrete model parameters. This is particularly relevant if the *dhfr* gene is to be included in future analyses. The computational feasibility of such analyses depends on the number of reported haplotypes, and on the number of full haplotypes used in the statistical model. We illustrate these ideas in electronic supplementary material, S5, through a case study based on molecular data collected from India [25], focusing on a *dhfr* + *dhps* quintuple mutation that is associated with clinical failure of SP [26]. There is also more work to do in extending the model to consider dependent GPs, as it is possible that the prevalences of different haplotypes are related to each other, using for example the linear model of coregionalization [27] or the semiparametric latent factor model [28].

At present, the World Health Organization provides recommendations for implementing intermittent preventive treatment in pregnancy with SP based on the prevalence of the *dhps* K540E and A581G mutations [29]. Although it is known that different *dhps* haplotypes confer different degrees of SP resistance [20], these recommendations are based on the prevalence of individual mutations, rather than that of full haplotypes. One possible reason is that there are no existing methods in the literature to infer the prevalence of full haplotypes from partially reported data. We address this gap in the literature by describing how a latent multinomial GP model can be used to produce spatio-temporal maps of these prevalences. The results we present in this paper are able to quantify the spread of various drug-resistant haplotypes, and provide uncertainty estimates that can help optimize sampling strategies for future monitoring of antimalarial drug resistance.

## Data Availability

The dataset supporting this article is available from the Wwarn repository: www.wwarn.org/tracking-resistance/sp-molecular-surveyor [30]. The code used for analysis is available from the Zenodo repository: https://zenodo.org/records/10354808 [31]. Further details of our methods and results are provided in electronic supplementary material [32].
